# Supplementary material for: How Morphological Computation Shapes Integrated Information in Embodied Agents
Source: Front Psychol. 2021 Nov 29;12:716433. doi: 10.3389/fpsyg.2021.716433 (PMC8666602; doi:10.3389/fpsyg.2021.716433)
Supplement: Supplementary file 1 [file Data_Sheet_1.PDF]

Supplementary Material

OPTIMIZATION USING THE EM-ALGORITHM

Here we are going to define the optimization using the em-algorithm. To simplify the notation we will refer to all the sampled distributions of the visible variables by

$$\hat{P}_{s,g|s,a} = \tilde{P}(s_t|a_t)\tilde{P}(s_{t+1}|s_t, a_t)\tilde{P}(s_{t+2}|s_{t+1}, a_{t+1})\tilde{P}(g|s_{t+2}, s_{t+1}, s_t, a_{t+2}, a_{t+1}, a_t). \tag{S1}$$

The em-algorithm iterates between two sets of distributions in order to find the minimal difference between them. In order to simplify the notation we will define $\mathcal{Y} = \mathcal{S} \times \mathcal{C} \times \mathcal{A}$ and $\mathcal{Z} = \mathcal{Y} \times \mathcal{Y} \times \mathcal{Y} \times \mathcal{G}$, such that $z = (s_t, c_t, a_t, s_{t+1}, c_{t+1}, a_{t+1}, s_{t+2}, c_{t+2}, a_{t+2}, g) \in \mathcal{Z}$. Let $\mathcal{P}(\mathcal{Z})$ be the set of probability distributions with the state space $\mathcal{Z}$ and let $\mathcal{P}^\circ(\mathcal{Z})$ consist of all the strictly positive distributions in $\mathcal{P}(\mathcal{Z})$. The first set we are considering is

$$\mathcal{M}_G := \{Q \in \mathcal{P}(\mathcal{Z})|Q(g = 1) = 1,\, Q(g = 0) = 0\}\,.$$

Every distribution in $\mathcal{M}_G$ achieves the goal with probability 1. This goal manifold is a linear family.

The second set consists of all the distributions that factor according to the architecture of the agents, meaning that each of these distributions describes a possible behavior of an agent.

$$\mathcal{M}_A := \Big\{P \in \mathcal{P}^\circ(\mathcal{Z})|P(z) = \hat{P}_{s,g|s,a}\, P(c_t|a_t, s_t)P(a_t) \prod_i P(a^i_{t+2}|s_{t+1}, c_{t+1}) \prod_i P(a^i_{t+1}|s_t, c_t) \prod_j P(c^j_{t+1}|s_t, c_t) \prod_j P(c^j_{t+2}|s_{t+1}, c_{t+1}), z \in \mathcal{Z}\Big\}$$

We will call $\mathcal{M}_A$ the agent manifold. Note that these two manifolds are disjoint, since every distribution in $\mathcal{M}_G$ has per definition values equal to zero and is therefore on the boundary of the probability simplex.

The difference between elements of these two manifolds will be calculated by using the KL-divergence.

DEFINITION 1 (KL-Divergence). *The Kullback-Leibler-divergence is defined as*

$$D(P \parallel Q) = \sum_{z \in \mathcal{Z}} P(z)\, log\left(\frac{P(z)}{Q(z)}\right)$$

with the conventions that $0 \cdot log\,\frac{0}{0} = 0,\ 0 \cdot log\,\frac{0}{Q(z)} = 0$ *and* $P(z) \cdot log\,\frac{P(z)}{0} = \infty$ *for* $P(z) > 0$.

This measures how much the uncertainty of the random variable increases, if we use Q instead of P. The KL-divergence has the following properties:

1. $D(P \parallel Q) \geq 0$

2. $D(P \parallel Q) = 0$ if and only if $P = Q$

Proofs of these properties can be found in (Cover and Thomas, 2006) in Theorem 2.6.3.

Using the em-algorithm we are able to find the minimal difference between these two manifolds

$$\inf_{P \in \mathcal{M}_A, Q \in \mathcal{M}_G} D(Q \parallel P).$$

Therefore this procedure results in the distribution $P \in \mathcal{M}_A$ that is closest to achieving the goal. The algorithm works by iteratively projecting to $\mathcal{M}_G$ with an e-projection, meaning minimizing the KL-divergence with respect to the first argument, and then projecting to $\mathcal{M}_A$ with an m-projection, defined by minimizing the KL-divergence with respect to the second argument. A sketch of this process is depicted in Figure S1.

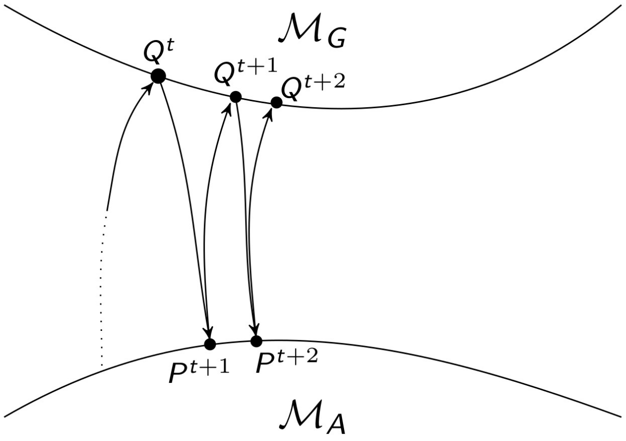

Figure S1. Sketch of the em-algorithm.

Let $P^0 \in \mathcal{M}_A$ be an arbitrary initial distribution and project this to $\mathcal{M}_G$ via an e-projection

$$Q^0 = \arg\inf_{Q \in \mathcal{M}_G} D(Q \parallel P^0).$$

Then we perform an m-projection to $\mathcal{M}_A$

$$P^1 = \arg\inf_{P \in \mathcal{M}_A} D(Q^0 \parallel P).$$

Repeating this leads to

$$Q^t = \arg\inf_{Q \in \mathcal{M}_G} D(Q \parallel P^t) \qquad P^{t+1} = \arg\inf_{P \in \mathcal{M}_A} D(Q^t \parallel P).$$

This em-algorithm is guaranteed to converge, but might converge towards a local minimum, see (Amari, 1995) or Section 5.3 of (Csiszár and Shields, 2004). In our setting, the local minima are also of interest as this gives us the opportunity to not only analyze one optimal behavior, but different strategies.

Now it remains to show what the projections are in our case. We will start with the e-projection. Projecting $P^t \in \mathcal{M}_A$ to a linear family w.r.t. the first variable is well known and can be performed in the following way

$$\begin{aligned} \arg\min_{Q \in \mathcal{M}_G} D(Q \parallel P^t) = Q^t \\ Q^t(z) = P^t(z) \cdot \frac{Q(g)}{P^t(g)} \end{aligned} \tag{S2}$$

Note that $Q(g)$ is the same for every element in $\mathcal{M}_G$ and that $P \in \mathcal{M}_A$ is strictly positive. Therefore this expression is well defined.

The m-projection can be performed as follows.

$$\begin{aligned} \arg\min_{P \in \mathcal{M}_A} D(Q^t \parallel P) =& P^{t+1} \\ P^{t+1}(z) =& Q^t(c_t|a_t, s_t)\hat{P}(s, g|s, a)Q^t(a_t) \prod_i Q^t(a^i_{t+1}|s_t, c_t) \\ & \prod_i Q^t(a^i_{t+2}|s_{t+1}, c_{t+1}) \prod_j Q^t(c^j_{t+1}|s_t, c_t) \prod_j Q^t(c^j_{t+2}|s_{t+1}, c_{t+1}). \end{aligned} \tag{S3}$$

Proofs of these projections can be found in the Proofs section. This algorithm is equivalent to the EM-algorithm used in statistics, see Section 8.1 in (Amari, 2016) or Section 5.3 in (Csiszár and Shields, 2004).

PROOFS

PROOF OF PROPOSITION 1. We write $y_t = (s_t, c_t, a_t) \in \mathcal{Y} = \mathcal{S} \times \mathcal{C} \times \mathcal{A}$,

$$\begin{aligned} & P(y_t, y_{t+1}) \\ & = \sum_{w_t, w_{t+1}} P(w_t) \cdot P(y_t|w_t) \cdot P(w_{t+1}|w_t, a_t) \prod_k P(s^k_{t+1}|w_{t+1}) \prod_i P(a^i_{t+1}|s_t, c_t) \prod_j P(c^j_{t+1}|s_t, c_t) \\ & = \prod_i P(a^i_{t+1}|s_t, c_t) \prod_j P(c^j_{t+1}|s_t, c_t) \sum_{w_t, w_{t+1}} P(w_t) \cdot P(y_t|w_t) \cdot P(w_{t+1}|w_t, a_t) \prod_k P(s^k_{t+1}|w_{t+1}) \end{aligned}$$

The sum describes

$$P(s_t, a_t, c_t, s_{t+1}) = \sum_{w_t, w_{t+1}} P(w_t) \cdot P(y_t|w_t) \cdot P(w_{t+1}|w_t, a_t) \prod_k P(s^k_{t+1}|w_{t+1}).$$

Now we take a closer look at $P(s_{t+1}|s_t, a_t, c_t)$ and show that S_{t+1} is independent of C_t given (S_t, A_t). For that we need to describe $P(y_t)$ in more detail. The graph corresponding to the distribution is a chain graph and therefore we are able to use Section 3.2.3 in (Lauritzen, 1996) to gain a finer parametrization.

There exist non-negative functions f_1, f_2, such that $P(s_t, a_t, c_t) = f_2(s_t, a_t, c_t) \sum_{w_t} f_1(s_t, w_t)$. Using this definition results in

$$\begin{aligned}
P(s_{t+1}|s_t, a_t, c_t) &= \frac{P(s_{t+1}, s_t, a_t, c_t)}{P(s_t, a_t, c_t)} \\
&= \frac{f_2(s_t, a_t, c_t) \sum_{w_t} f_1(s_t, w_t) \sum_{w_{t+1}} P(w_{t+1}|w_t, a_t) \prod_k P(s_{t+1}^k|w_{t+1})}{f_2(s_t, a_t, c_t) \sum_{w_t} f_1(s_t, w_t)} \\
&= \frac{\sum_{w_t} f_1(s_t, w_t) \sum_{w_{t+1}} P(w_{t+1}|w_t, a_t) \prod_k P(s_{t+1}^k|w_{t+1})}{\sum_{w_t} f_1(s_t, w_t)} \\
&= P(s_{t+1}|s_t, a_t)
\end{aligned}$$

Therefore the factorization of P can be written as

$$P(y_t, y_{t+1}) = P(y_t) \cdot \prod_i P(a_{t+1}^i|s_t, c_t) \prod_j P(c_{t+1}^j|s_t, c_t) \cdot P(s_{t+1}|s_t, a_t).$$

PROOF OF (S2). In order to proof that Q^t is the e-projection of P to $\mathcal{M}_G$ we make use of the log-sum inequality and the convention that $0 \cdot log\, 0 = 0$. Let $Q \in \mathcal{M}_G$ then

$$\begin{aligned}
D(Q \parallel P^t) &= \sum_z Q(z)\, log \left(\frac{Q(z)}{P^t(z)} \right) \\
&\geq \sum_g \left(\sum_{y_t, y_{t+1}, y_{t+2}} Q(z) \right) log \left(\frac{\sum_{y_t, y_{t+1}, y_{t+2}} Q(z)}{\sum_{y_t, y_{t+1}, y_{t+2}} P^t(z)} \right) \\
&= 1 * log \left(\frac{1}{P^t(g)} \right) + 0 * log \left(\frac{0}{P^t(g)} \right) \\
&= \sum_{y_t, y_{t+1}, y_{t+2}} P^t(z) \frac{1}{P^t(g)} * log \left(\frac{1 \cdot P^t(z)}{P^t(g) P^t(z)} \right) \\
&= D(Q^t \parallel P^t)
\end{aligned}$$

PROOF OF (S3). The KL-divergence between $Q^t \in \mathcal{M}_A$ and $P \in \mathcal{M}_G$ can be written as

$$
\begin{aligned}
D(Q^t \parallel P) &= \sum_z Q^t(z)\, log\, \frac{Q^t(z)}{P(z)} \\
&= \sum_z Q^t(z)\, log\, \frac{Q^t(z)}{\hat{P}(s,g|s,a)} + \sum_z Q^t(z)\, log\, \frac{1}{P(c_t|a_t,s_t)} + \sum_z Q^t(z)\, log\, \frac{1}{P(a_t)} \\
&+ \sum_z Q^t(z)\, log\, \frac{1}{\prod_i P(a^i_{t+1}|s_t,c_t)} + \sum_z Q^t(z)\, log\, \frac{1}{\prod_i P(a^i_{t+2}|s_{t+1},c_{t+1})} \\
&+ \sum_z Q^t(z)\, log\, \frac{1}{\prod_j P(c^j_{t+1}|s_t,c_t)} + \sum_z Q^t(z)\, log\, \frac{1}{\prod_j P(c^j_{t+2}|s_{t+1},c_{t+1})}
\end{aligned}
.
$$

The last six sums are called cross-entropies. It follows directly from the definition of the KL-divergence and property 1. that the cross-entropy is greater or equal to entropy. Therefore we gain the following inequality.

$$
\begin{aligned}
D(Q^t \parallel P) \geq &\sum_z Q^t(z)\, log\, \frac{Q^t(z)}{\hat{P}(s,g|s,a)} + \sum_z Q^t(z)\, log\, \frac{1}{Q^t(c_t|a_t,s_t)} + \sum_z Q^t(z)\, log\, \frac{1}{Q^t(a_t)} \\
&+ \sum_z Q^t(z)\, log\, \frac{1}{\prod_i Q^t(a^i_{t+1}|s_t,c_t)} + \sum_z Q^t(z)\, log\, \frac{1}{\prod_i Q^t(a^i_{t+2}|s_{t+1},c_{t+1})} \\
&+ \sum_z Q^t(z)\, log\, \frac{1}{\prod_j Q^t(c^j_{t+1}|s_t,c_t)} + \sum_z Q^t(z)\, log\, \frac{1}{\prod_j Q^t(c^j_{t+2}|s_{t+1},c_{t+1})} \\
&= D(Q^t \parallel P^{t+1})
\end{aligned}
$$

REFERENCES

Amari, S. (2016). *Information Geometry and Its Applications* (Japan: Springer)

Amari, S.-i. (1995). Information geometry of the em and em algorithms for neural networks. *Neural Networks* 8, 1379–1408. doi:https://doi.org/10.1016/0893-6080(95)00003-8

Cover, T. M. and Thomas, J. A. (2006). *Elements of Information Theory (Wiley Series in Telecommunications and Signal Processing)* (USA: John Wiley & Sons, Inc.)

Csiszár, I. and Shields, P. (2004). Information theory and statistics: A tutorial. *Foundations and Trends® in Communications and Information Theory* 1, 417–528. doi:10.1561/0100000004

Lauritzen, S. L. (1996). *Graphical Models* (Oxford: Clarendon Press)
